# Novel Pestiviruses Detected in Cattle Interfere with Bovine Viral Diarrhea Virus Diagnostics

**DOI:** 10.3390/v16081301

**Published:** 2024-08-15

**Authors:** Judith Köster, Karla Schneider, Dirk Höper, Andreas Salditt, Martin Beer, Thomas Miller, Kerstin Wernike

**Affiliations:** 1 Aulendorf State Veterinary Diagnostic Centre, Löwenbreitestraße 18/20, 88326 Aulendorf, Germany; 2 Institute of Diagnostic Virology, Friedrich-Loeffler-Institut, Südufer 10, 17493 Greifswald-Insel Riems, Germany

**Keywords:** pestivirus, ruminants, cattle, diagnostics, control, epidemiology, sequence analysis

## Abstract

Since the start of the mandatory nationwide bovine viral diarrhea (BVD) eradication program in Germany in 2011, the number of persistently infected (PI) animals has decreased considerably, resulting in a continuous decrease in seroprevalence. The increasingly BVD-naive cattle population could facilitate spillover infections with non-BVDV ruminant pestiviruses. Here, we report two cases in which novel pestiviruses were isolated from cattle; in both cases, the whole genome sequence showed the highest level of identity to strain “Pestivirus reindeer-1”. Both novel viruses gave positive results in BVDV diagnostic test systems, confirming that cross-reactivity is an important issue in pestivirus diagnostics. In the first case, the pestivirus was probably transmitted from sheep kept with the affected cattle, suggesting that the co-housing of small ruminants and cattle is a risk factor. The source of infection could not be determined in the second case. The occurrence of these two cases in independent cattle holdings within a relatively short time frame suggests that it would be useful to determine the presence of pestiviruses in small ruminants or even wild ruminants to better assess risk factors, especially for BVDV-free populations.

## 1. Introduction

The genus *Pestivirus* of the family *Flaviviridae* includes four classical species, namely *Pestivirus bovis* (formerly known as bovine viral diarrhea virus (BVDV)-1), *Pestivirus tauri* (BVDV-2), *Pestivirus ovis* (border disease virus (BDV)) and *Pestivirus suis* (classical swine fever virus (CSFV)). Additionally, other pestiviruses have been described in various species [1]. For ruminants, BVDV and BDV are of particular interest. Bovine pestiviruses can be transmitted between different animal species, including cattle, sheep, goats, and various wildlife animals [2,3]. BVDV can cause severe diseases, predominantly in cattle, and is responsible for significant economic losses worldwide [4]. An infection with BVDV has various clinical manifestations, from asymptomatic infections to mild unspecific clinical signs such as fever, diarrhea, or pneumonia and, in addition, immunosuppression, which can lead to further infections [5]. The consequences of infections of pregnant cows vary depending on the stage of pregnancy. Fetal resorptions, abortions, stillbirths, or the birth of weak or malformed calves may occur [6]. Infections at between 30 and approximately 90 days of gestation can result in the birth of persistently infected (PI) animals [7]. These PI animals can either develop normally with no clinical signs or suffer from mucosal disease, which causes high fever, anorexia, and diarrhea and leads to death within days to weeks [5,6]. PI animals pose the highest risk of infecting other animals because they permanently shed large amounts of infectious virus [8].

BDV also occurs worldwide and has an impact on reproduction in sheep and therefore a noteworthy economic impact [9]. In Germany, the isolation of BDV in sheep, wild ruminants, and cattle has been reported [10,11]. BDV can cause clinical signs like abortion, stillbirths, and the birth of lambs with hairy fleeces and tremors (“hairy shaker syndrome“) [9,12]. Although BDV is mainly associated with the infection of small ruminants, it can also infect cattle [13,14,15,16]. In acute BDV infections in cattle, mild non-specific clinical signs have been reported, but in most cases, infected animals showed no changes at all [13]. Furthermore, several cases of persistently BDV-infected cattle have been reported, and these PI animals showed clinical signs like diarrhea and wasting [14,17]. The highest risk of an infection with BDV or related viruses for cattle is direct contact with infected sheep when housed together or when grazing on the same pasture [14,18]. The transmission of BDV between cattle is also possible, as described in the case of an infected bull in New Zealand and in a study where BDV was transmitted to heifers from a persistently infected calf [13,15]. The role of wildlife populations in the transmission of ruminant pestiviruses is also discussed. It was shown that wild ruminants, but also other wild animals like the European hare, are susceptible to pestiviruses, meaning that wild animals could be a potential source of infection for cattle [19,20,21]. However, in Nordic countries, no direct influence on cattle populations was observed despite high pestivirus seroprevalences in wild ruminants, presumably due to the limited contact between the species [22]. At the same time, it was pointed out that the risk of transmission between cattle and wild ruminants depends on various evolving factors and should be further investigated [23]. 

Since 2011, a nationwide mandatory BVDV eradication program has been in place in Germany. In order to detect PI animals, all newborn calves have to be tested for BVDV during their first days of life. The vast majority of animals is tested through ear notch samples taken during the tagging process. To detect infected animals, antigen ELISA or real-time RT-PCR are typically used. Distinguishing between BVDV and BDV can be crucial for suitable control measures and effective surveillance. However, the close genetic and antigenetic relationship and, therefore, the cross-reactivity between these viruses often pose a challenge in differentiating them [24]. The available techniques, like discriminating RT-qPCRs, sequencing, or the differential staining of virus isolates using species-specific monoclonal antibodies, are laborious and time-consuming [24,25]. This also applies for the neutralization assay, the gold standard for serological diagnostics, with which detected antibodies can be differentiated despite cross-reactivity [26]. Since the start of the compulsory eradication program in 2011, the prevalence of PI animals in relation to the number of newborn calves has been continuously decreasing throughout Germany [27]. In the federal state of Baden-Württemberg (BW), the last BVDV PI animal was reported in 2020. Since then, no case of BVD has occurred in BW, and, on this basis, the disease-free status was approved according to the Commission Implementing Regulations (EU) 2021/620 and 2022/1218. In 2023, two ear notch samples collected from different cattle holdings were detected as reactive in routine BVDV testing, the first sample in April and the second sample in September. Both cases are described below.

## 2. Materials and Methods

### 2.1. Cattle Farms and Diagnostic Samples

#### 2.1.1. Herd 1

The first case occurred in April 2023; the affected male calf originated from a farm with suckler cows. The 18 suckler cows were kept together with 19 calves; 10 of them were born on the farm. Fifty beef cattle were housed in a separate stable, and about 400 sheep were kept on the farm. There was no direct contact between the cattle and the sheep, but leftover food from the sheep was fed to the cattle. The affected calf tested positive through antigen ELISA in the course of routine ear notch testing for BVDV surveillance. For further clarification, a second ear notch sample and a blood sample were taken from the calf as additional follow-up samples seven days after the first sampling. Six days later, the calf was euthanized and examined pathologically; tissue from the thymus, intestines, and a pool of different organs was preserved for further examination. Furthermore, at the same time as the calf’s follow-up samples were collected, blood samples were taken from 20 cattle that were kept in direct contact with the calf, including the calf’s dam. In addition, 40 sheep were sampled during the investigations, including ewes, lambs, and purchased animals of different ages. 

#### 2.1.2. Herd 2

The second farm was a dairy cow farm. At the time of the case in September 2023, the farm had 252 animals. Of these, about 140 were dairy cows, and 124 were lactating. There were also 64 breeding cattle and heifers and 48 calves on the farm. Some heifers, including the mother of the calf of interest, were kept on a pasture during their pregnancy. The animals escaped from the pasture for approximately 12 h. During this period, the whereabouts and potential contact of the dam with small ruminants, wild ruminants, or other animals are unknown. The escape occurred when the calf’s mother was at the beginning of the second trimester of pregnancy. As described in the first case, the ear notch sample of the calf of the aforementioned heifer also tested positive in the antigen ELISA during BVDV routine testing. At the time of the first positive test result, one week after the female calf was born, the animal had already died. A pathological examination was carried out, and an additional skin sample, blood sample, and tissue from the intestines and a pool of different organs were collected. For epidemiologic reasons and to monitor the dynamics of the infection, additional animals on the farm were also sampled. Two days after the death of the calf, blood samples were collected from 68 animals of the herd, and two weeks later, blood samples from all animals in the herd as well as individual milk samples from 124 lactating cows and one bulk milk sample were collected. It should be emphasized that the calf´s mother, two cows that were in the same stable when the calf was born, and a calf that was kept in the immediately adjacent calf igloo were included in the sampling.

### 2.2. Virus Detection by Antigen ELISA, Real-Time RT-PCR, and Virus Isolation

Serum and ear notches were tested for pestivirus antigen (Ag) using the commercially available test kit ”BVDV Ag/Serum Plus Test“ (IDEXX, Liebefeld, Switzerland) according to the manufacturer’s instructions.

Nucleic acid was extracted from blood and tissue samples by using the IndiMag Pathogen Kit (Indical, Leipzig, Germany) in combination with the IndiMag 48s extraction instrument (Indical). Real-time RT-PCR was performed with the commercially available test kit ”virotype BVDV 2.0 RT-PCR Kit” (Indical) and a previously published generic panpesti real-time RT-PCR [28]. A positive control, a no-template control, and a negative control were included according to the instructions. Additionally, the commercial kit contained two endogenous controls (IC and beta actin). A CFX96 Thermocycler (Bio-Rad, Hercules, CA, USA) was used for amplification.

#### 2.2.1. Virus Isolation

Virus isolation was carried out from various tissues from both calves. Two different cell lines, MDBK and SFT-R cells (thymus, domestic sheep (ovis orientalis aries)), were used (L0261 and L0043, Collection of Cell Lines in Veterinary Medicine, Insel Riems, Germany). Cells were maintained in E-MEM (Biowest, Nuaillé, France) supplemented with 10% fetal calf serum (FCS, Biowest, Nuaillé, France), 64 µg/mL penicillin and 100 µg/mL streptomycin (Sigma-Aldrich, Steinheim, Germany), 1% sodium pyruvate (Bio&SELL GmbH, Feucht, Germany), and 1% non-essential amino acids (Biowest, Nuaillé, France). During virus propagation, no FCS was used. The cells and medium were free of pestiviruses, as tested by real-time RT-PCR during the studies. FCS was also free of antibodies against pestiviruses, which was confirmed by the virus neutralization assay (VNT). Five grams of the tissue samples were shredded, and phosphate-buffered saline (PBS) was added to produce a 10% suspension. After homogenization and one-hour storage at 6 °C, the samples were centrifuged, and the supernatant was used as inoculum. The cell cultures were covered with 0.5 mL of the prepared samples in 24-well plates. The inoculated cells were incubated for 1 h at 37 °C with 5% CO_2_, and then the sample material was removed. The cells were washed with 1 mL PBS, which was then discarded, and the same amount of FCS- and sodium pyruvate-free medium as described above was added. The inoculated cells were incubated at 37 °C with 5% CO_2_ for 5 days and checked daily for cell toxic and cytopathic effects. The virus strains BVDV-1 NADL and BDV 137/4 (virus collection, Insel Riems, Germany) were included as a positive control, and pure cells and medium were included as negative controls. Two passages were carried out and stained after 5 days using peroxidase staining protocols for pestivirus detection. For this, the maintenance medium was removed, and the cells were washed with PBS and then heat-fixated for 1.5 h at 80 °C. The pestivirus group-specific monoclonal antibody WB103/105 (diluted 1/100 in PBS, APHA Scientific, Weybridge, UK) was added to each well and incubated for 1 h at room temperature in a humidified chamber. Thereafter, the plates were emptied and washed, and polyclonal anti-mouse IgG HRP (Agilent Technologies Denmark ApS, Glostrup, Denmark) was added to each well. After incubating for 1 h at room temperature in a humidified chamber and washing, 0.3 mL of substrate solution (3,3′-Diaminobenzidine and Cl_2_Ni, Sigma-Aldrich, Steinheim, Germany) was added to each well. After 10 min of incubation in the dark at room temperature, the staining was assessed with a Nikon Eclipse Ts2-FL inverse fluorescence microscope.

#### 2.2.2. Immunofluorescence Staining

The viruses isolated from the affected calves were passaged once in the SFT-R cells. Thereafter, immunofluorescence staining was performed using SFT-R cells infected with either isolate as an antigen matrix. As a control, uninfected SFT-R cells were used. In brief, the cells were infected and incubated for 72 h at 37 °C in a 5% CO_2_ atmosphere. Thereafter, the cell culture supernatant was removed, and the cells were fixated by heat (80 °C, 2 h). Subsequently, either the pestivirus group-specific antibodies WB103/105 (diluted 1/500 in Tris-buffered saline with Tween 20 (TBST)), the BVDV-1-specific monoclonal antibody WB215 (diluted 1/100 in TBST), the BVDV-2-specific monoclonal antibody WS538 (diluted 1/100 in TBST), or the BDV-specific monoclonal antibody WS363 (diluted 1/100 in TBST) (all APHA Scientific, Weybridge, UK) was added and incubated for 1 h. Following a washing step with TBST, the cells were incubated with an FITC-labeled polyclonal goat anti-mouse antibody (Agilent Technologies Denmark ApS, Glostrup, Denmark) for 1 h. Finally, the cells were washed again, and a DABCO (1,4-diazabicyclo [2.2.2]octan, Sigma-Aldrich, Steinheim, Germany) fluorescence preservation buffer combined with propidium iodide (Sigma-Aldrich, Steinheim, Germany), to stain the cell nuclei, was added. The plates were assessed using a Nikon Eclipse Ti-S inverse fluorescence microscope.

### 2.3. Sequencing and Sequence Analysis

For full-genome sequencing, RNA was extracted from the culture-grown viruses after disintegration using the Covaris cryoPREP CP02 Impactor (Covaris Inc., Woburn, MA, USA), as described by Wylezich and colleagues [29]. For the lysis of the disintegrated sample material, lysis buffer from the RNAdvance Tissue Kit (Beckman Coulter, Krefeld, Germany) was used, and extraction was performed using the same kit and a KingFisher FLEX instrument (Thermo Fisher Scientific, Darmstadt, Germany), including a DNase Treatment (RNase-Free DNase, Qiagen, Hilden, Germany). Subsequently, RNA was concentrated using RNAClean XP Beads (Beckman Coulter) and then quantified with an Implen N60 instrument (Implen, Munich, Germany). After reverse transcription of the complete available RNA (~60 ng) using a SuperScript IV First-Strand cDNA Synthesis System (Thermo Fisher), second strand synthesis was performed with the NEBNext Ultra II Non-Directional RNA Second Strand Synthesis Module (New England Biolabs, Frankfurt am Main, Germany). The generated double-stranded DNA was fragmented with a Covaris M220 Focused ultrasonicator (Covaris), then purified with Agencourt AMPure XP Beads (Beckman Coulter), and the purified fragmented DNA was used for library preparation with an NEBNext Fast DNA Library Prep Set for Ion Torrent (New England Biolabs). After size selection with Agencourt AMPure XP Beads (Beckman Coulter), library quality was checked with an Agilent High Sensitivity DNA assay on an Agilent BioAnalyzer 2100 (both Agilent, Waldbronn, Germany), and subsequently, the library was quantified with the aid of a QIAseq Library Quant Assay Kit for Ion Torrent (Qiagen). Finally, the libraries were pooled and prepared for sequencing with an Ion Chef instrument and sequenced with an Ion Torrent S5 XL on an Ion 530 Chip with an Ion 510 & Ion 520 & Ion 530 Kit—Chef (all Thermo Fisher Scientific). Raw data were trimmed of remaining adapter sequences and low-quality regions and assembled with t454 Newbler software (v3.0; Roche, Mannheim, Germany). The sequences generated in this study were submitted to the International Nucleotide Sequence Database Collaboration databases (GenBank accession numbers PP812599 and PP812600). The newly generated sequences were compared to sequences of pestivirus reference strains obtained from NCBI GenBank by Geneious prime version 2021.0.1 (Biomatters, Auckland, New Zealand). Based on the alignment, a Neighbor-Joining tree was calculated using the Tamura 3-parameter method with 500 bootstrap replicates through MEGA X software (version 10.2.4) [30,31].

### 2.4. Antibody Detection by ELISA and Virus Neutralization Test (VNT)

Serum and milk samples were tested by the commercially available ID Screen BVD p80 Antibody Competition ELISA (Innovative Diagnostics, Grabels, France) for antibodies against the p80 (NS3) protein. The testing and evaluation of the results were carried out according to the instructions of the manufacturer. Positive and negative controls were included as recommended, and two confirmed positive samples and one confirmed negative sample were tested in each run as secondary controls. The optical density was measured with a photometer (Sunrise TM, Tecan, Switzerland).

A virus neutralization test was performed to quantify and differentiate the antibody-positive samples. After the inactivation of the sera for 30 min at 56 °C, a two-fold serum dilution series was prepared with a starting dilution of 1/5. A virus suspension was added to each well, which was previously titrated and adjusted to 100 tissue culture infective doses (TCID) per 50 µL. The following viruses were applied: BVDV-1 strain NADL, BVDV-2 strain CS8644, BDV strain Moredun, BDV strain 137/4, and the isolate from the first case in April 2023 (isolate ID: VI 958 2023BVD03439). After 2 h at 37 °C and 5% CO_2_, 100 µL of a suspension of cells was added. The plates were then incubated at 37 °C for 4 days. Virus growth was detected by peroxidase staining as described above, and the titer was calculated by using the method of Spearman–Kärber [32,33]. In order to differentiate the antibodies, the titers determined for each virus strain were compared, and an at least four-fold higher titer was necessary for the assignment, as recommended in the manual of diagnostic tests and vaccines for terrestrial animals [34].

## 3. Results

### 3.1. Clinical and Pathological Findings and Virus Detection

No clinical or pathological changes were observed in the first infected calf. The second calf showed clinical signs of respiratory disease, and the pathological examination revealed alterations suggestive of aspiration pneumonia as well as enterocolitis.

The ear notch samples from both calves were clearly positive in the antigen ELISA (corrected optical density (OD) of 2.5 in the first case and 3.6 in the second case; test cut-off for positivity > 0.3). In order to confirm the results and investigate the cause of the outbreak, follow-up blood samples were taken from both calves. The results of the ear notch samples were confirmed, as the blood samples also tested positive in the antigen ELISA (OD 1.5 and 1.6, respectively). Since the beginning of the mandatory BVDV eradication program in 2011, all previous ear notch samples have tested negative in both farms. Pestivirus RNA was detected in the blood sample from the first calf using the commercial real-time RT-PCR with a quantification cycle (Cq) value of 38.7 and by a panpesti RT-qPCR with a Cq value of 32.5. In the samples taken at autopsy, pestivirus RNA could likewise be detected. The thymus and the skin of the first calf, the skin and intestines of the second calf, and a pool of different organs from both calves tested positive in the commercial BVDV real-time RT-PCR kit. Overall, the Cq values ranged between 32.3 and 39.5, with the Cq value of the skin of the second calf being the lowest at 32.3. For both ear notches as well as the skin, intestines, and a pool of different organs from the second calf, lower Cq values were detected with the additional panpesti RT-PCR (Cq values from 14.0 to 31.3).

All the other animals examined, the cows from both farms and the sheep, showed negative results in the antigen ELISA, as well as the commercial real-time RT-PCR. An infectious virus could be isolated from the calf´s thymus in the first case, from the calf´s intestines in the second case, and from a pool of different organs in both cases. The isolated virus strain from the first case is further referred to as “VI 958 2023BVD03439”, and for the second case, it is referred to as “VI 1861 2023BVD08322”. Virus isolation was not successful from the blood or from the skin.

### 3.2. Virus Characterization

**Figure 1:**
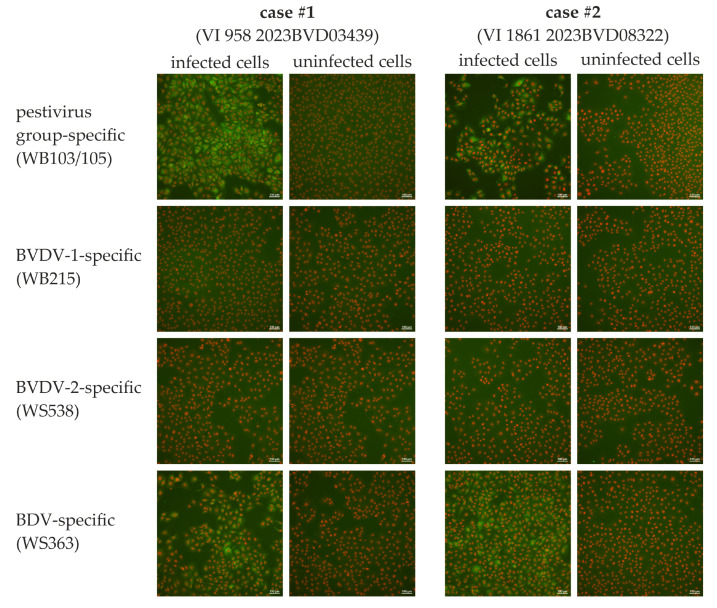
Immunofluorescence staining of SFT-R cells infected with the virus isolates from both newborn calves or uninfected cells using the indicated monoclonal antibodies. Antibodies against pestiviruses are stained green, and cell nuclei appear in red. Scale bars indicate 100 µm.

Both virus isolates could be stained by immunofluorescence methods using pestivirus group-specific and BDV-specific monoclonal antibodies. No fluorescence signal could be seen when applying BVDV-1-specific or BVDV-2-specific antibodies (Figure 1).

**Figure 2:**
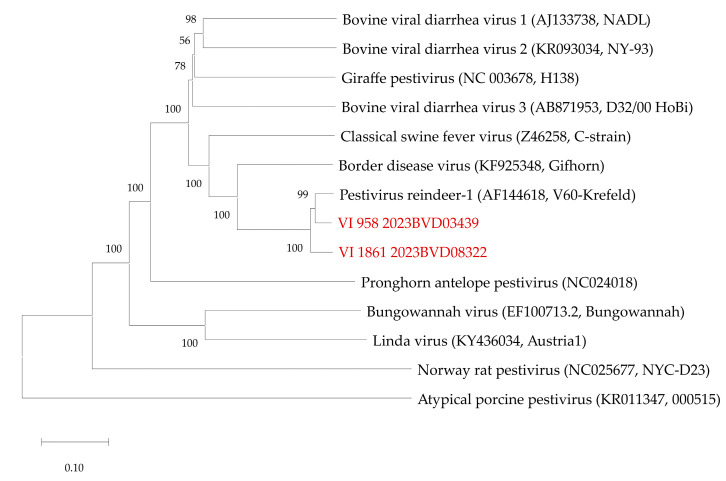
Phylogenetic relation of the virus isolates generated in the present study (red) to other pestiviruses. The phylogenetic tree is based on complete coding sequences. For the reference strains, INSDC GenBank accession numbers and strain names are given in brackets. Scale bar indicates nucleotide substitutions per site.

Complete genome sequences were generated from both virus isolates. At the nucleotide level, the sequences showed an identity of 93.02% to each other and of 94.88% (first case) and 93.66% (second case), respectively, to the most closely related sequence, which belongs to the Pestivirus strain “Reindeer-1” (GenBank accession number AF144618) (Figure 2).

### 3.3. Serological Investigations of the Affected Farms

Both calves were tested for antibodies against pestiviruses by the ELISA. The first calf was seropositive (S/N value of 6.2%; test cut-off for positivity ≤ 40%), while the second calf was seronegative (S/N value of 58.4%) (Table 1). Additional animals were sampled on both farms, as indicated in the methods section, and in both cases, further seropositive animals were found. In the first case, antibodies could be detected in the sera of 16 out of 19 sampled animals, while in the second case, only three of the 252 animals were seropositive, with one of the cows seroconverting between the first and second examination. For all seropositive animals, direct contact with the infected calf was reported. The examination of the sheep from the first farm identified one doubtful and 30 seropositive sheep out of 40 sampled animals. In the seropositive sheep, all age groups were represented with S/N values from 4.9 to 28.7%, while the animal with the doubtful result was an ewe with an S/N value of 47.1%.

In the VNT, the neutralization of different ruminant pestiviruses by selected serum samples from the seropositive cows and sheep was further examined in order to differentiate the detected antibodies. The determined neutralizing titers tended to be higher against the used BDV strains than against the BVDV strains. However, markedly higher titers (at least a four-fold titer difference) could only be measured when using the virus isolated from the first calf’s organs (Table 2).

In addition to the sera, milk samples taken at the second farm were analyzed using the commercial antibody ELISA. The results of the individual milk samples were consistent with those of the corresponding blood tests; the milk of only two of the lactating cows tested positive. The bulk milk sample contained milk from 124 lactating cows and tested negative, but with an increased value (S/N: 69.3%, test cut-off for bulk milk and seroprevalence of ≥2%: ≤65%) in comparison to bulk milk samples taken for BVDV antibody status determination at this farm in 2022 (S/N: 96.1%, 87.6%) and 2023 (S/N: 88%, 108.8%).

## 4. Discussion

The prevalence of BVDV in cattle in Germany is steadily decreasing due to the successful control of BVDV through ear notch testing and the elimination of PI animals [27]. The occurrence of two cases of novel pestiviruses in cattle within one year indicates that such cases are favored by the declining seroprevalence. In both cases, the calves were identified as being infected with a pestivirus by BVDV test systems, which supports previous observations that cross-reactivity is an important issue in pestivirus diagnostics [25]. Especially when BVDV control programs are in place, the subsequent typing of detected viruses is strongly recommended to differentiate BVDV from BDV and further ruminant pestiviruses and to support suitable control measures [24]. When using serological methods, differentiation between antibodies is only enabled by the parallel application of neutralization tests against isolates of the different pestivirus species, which requires isolation in cell culture when new viruses emerge [26]. However, previous studies have shown that despite using different virus strains, there can still be samples that cannot be assigned to a certain virus species [16]. In these cases, sequencing of the viral genomes is necessary for a final diagnosis.

The fact that two independent cases of novel pestiviruses in cattle occurred within a short period of time raises the question of the source of transmission and potential risk factors. The first calf was born at a suckler cow farm where about 400 sheep are also kept. The pestivirus was isolated from the calf´s tissue, and antibodies were detected. Since the calf ingested colostrum from its mother, it is most likely that the seropositivity of the calf was caused by maternal antibodies. There was no evidence of acute virus circulation from the examination of the other animals; no virus genome was detected in either the sheep or the cows, but a high proportion of the sampled animals, 85.0% of the cows and 77.5% of the sheep, had antibodies that could be identified as antibodies against the newly isolated pestivirus strain by using VNTs. Since the farm fed leftover feed from the sheep to the cows, but no direct contact was reported, it could be assumed that the virus was indirectly transmitted from the sheep to the cows, thus explaining the high seroprevalence. The possibility of interspecies transmission of ruminant pestiviruses has been previously demonstrated by several cases of BDV transmission from sheep to cattle [3,35]. Further, the transmission of the ovine BDV between cattle is also possible [15]. Therefore, common housing of small ruminants, especially sheep, and cattle poses a considerable risk for the infection of cattle with BDV [18]. Aside from transmission between different species, the emergence of new pestiviruses in cattle has also been described [36]. The possible origin of novel pestiviruses from wild animals, such as bats, and the unknown effects on animal health represent a risk that should not be underestimated [37].

As in the first case, the virus could be isolated from the second calf´s tissue. However, no antibodies could be detected in the blood sample of the calf, which could indicate that the calf was a PI animal. The absence of maternal antibodies in this case is likely due to the common practice on many dairy farms of separating the calf from the dam very early, which is why the calf may not have ingested the dam´s colostrum. Another reason could be that the calf, being already weak, did not ingest enough colostrum overall. Among the remaining animals in the herd, only a few that came into direct contact with the calf were seropositive, and all of them tested negative for the viral genome. In the second case, the source of the infection could not be identified, but an infection might have occurred when the dam escaped from the pasture. The fact that there were only a few other seropositive animals in the second herd hints at direct contact between only a small number of animals and the infected calf. The genome sequence of this virus is most closely related to a virus strain isolated from a reindeer at the Duisburg Zoo in 1996 suffering from severe diarrhea and anorexia [10]. In a further examination, eleven other animals from the herd were serologically positive, which indicates the circulation of the virus in the herd [38]. The description of the susceptibility of wild ruminants to a closely related virus strain suggests that in this case wild ruminants could be likewise susceptible to the isolated pestivirus and thus a potential source of infection for further livestock animals.

As described above, while the first calf showed no changes at all, the clinical signs and pathological changes in the second calf did not clearly indicate an infection with pestiviruses. In addition to asymptomatic cases, mild fever has previously been reported in cattle acutely infected with BDV [14], but as body temperature was not measured in the calves described in this study, such mild signs could have been overlooked. Acute infections with BVDV usually do not cause clinical signs, but mild diarrhea, respiratory symptoms, and immunosuppression can occur [5]. Cattle persistently infected with BDV might show similar clinical signs to BVDV PI cattle; diarrhea, wasting, and a weak-born calf have been reported [17]. However, in the two cases described here, there was no typical clinical manifestation of an infection with a pestivirus in the cattle. Nevertheless, since BVDV and BDV infections cannot be distinguished with certainty based on clinical signs, laboratory tests are necessary in any case, and these tests confirmed the presence of a virus most closely related to Pestivirus reindeer-1 in both newborn calves.

## 5. Conclusions

The occurrence of two cases of novel pestiviruses in cattle in Southern Germany within a short period of time shows the challenges posed by declining seroprevalences during the final phases of an ongoing BVD eradication program. The increasingly BVDV-seronegative cattle population appears to be highly susceptible to further, possibly hitherto unknown, pestiviruses. The possible paths of infection are manifold and cannot always be determined with certainty. For future BVDV diagnostics and control measures adapted to changing conditions, the seroprevalence of pestiviruses in small ruminants and their occurrence in wild animals should be examined more closely, and other possible risk factors should be identified. The adaptation and circulation of such pestiviruses in the BVDV-naive cattle population in Germany must be prevented, e.g., by early detection and characterization.

## Figures and Tables

**Table 1:** Results of viral antigen, genome, and antibody detection for ear notches and blood samples for both calves. n.t.—not tested.

Sample	Antigen ELISA (S-N Value)	Commercial BVDV RT-qPCR (Cq Value)	Panpesti RT-qPCR (Cq Value)	Antibody ELISA (S/N Value in %)
Calf case #1—ear notch	2.5 (positive)	n.t.	27.7 (positive)	n.t.
Calf case #1—blood	1.5 (positive)	38.5 (positive)	32.5 (positive)	6.2 (positive)
Calf case #2—ear notch	3.6 (positive)	n.t.	17.7 (positive)	n.t.
Calf case #2—blood	1.6 (positive)	no Cq (negative)	n.t.	58.4 (negative)

**Table 2:** Overview of the results of the serum samples selected for the VNT. The cut-off values for the antibody ELISA were ≥50% negative, between 40% and 50% doubtful, and ≤40% positive. For the neutralization tests (VNT), different ruminant pestiviruses including the newly generated isolate from the first case (VI 958 2023BVD03439) were used. Negative stands for a detection limit of <1/5 in the VNT. n.t.—not tested, n.d.—not defined.

Sample	Antibody ELISA (S/N Value in %)	VNT Titer against			Quotient of BVDV NADL and	
		BVDV NADL	BDV 137/4	Pestivirus Strain Case#1 (VI 958 2023BVD03439)	BDV 137/4	Pestivirus Strain Case#1 (VI 958 2023BVD03439)
**Case 1**						
Mother of calf 1	4.9	480	960	5120	2	11
Calf 1	6.2	40	240	n.t.	6	n.d.
Sheep (farm 1)	7.9	20	80	960	4	48
Sheep (farm 1)	5.7	240	960	5120	4	21
Sheep (farm 1)	12.4	30	480	2560	16	85
Sheep (farm 1)	4.9	60	160	3840	3	64
Sheep (farm 1)	7.4	20	80	920	4	46
Sheep (farm 1)	5.0	120	640	5120	5	43
Sheep (farm 1)	5.7	320	960	5120	3	16
Sheep (farm 1)	11.9	10	230	1280	23	128
**Case 2**						
Mother of calf 2	7.2	240	1280	5120	5	21
Cow (farm 2)	24.5	negative	negative	40	n.d.	40
Cow (farm 2)	35.1	negative	negative	5	n.d.	5

## Data Availability

The sequences generated and analyzed for this study were submitted to the International Nucleotide Sequence Database Collaboration databases (www.insdc.org) (GenBank accession numbers PP812599 and PP812600).

## References

[1] Postler T.S., Beer M., Blitvich B.J., Bukh J., de Lamballerie X., Drexler J.F., Imrie A., Kapoor A., Karganova G.G., Lemey P. (2023). Renaming of the genus *Flavivirus* to *Orthoflavivirus* and extension of binomial species names within the family *Flaviviridae*. Arch. Virol..

[2] Passler T., Walz P.H. (2010). Bovine viral diarrhea virus infections in heterologous species. Anim. Health Res. Rev..

[3] Becher P., Orlich M., Shannon A.D., Horner G., König M., Thiel H.J. (1997). Phylogenetic analysis of pestiviruses from domestic and wild ruminants. J. Gen. Virol..

[4] Richter V., Lebl K., Baumgartner W., Obritzhauser W., Käsbohrer A., Pinior B. (2017). A systematic worldwide review of the direct monetary losses in cattle due to bovine viral diarrhoea virus infection. Vet. J..

[5] Lanyon S.R., Hill F.I., Reichel M.P., Brownlie J. (2014). Bovine viral diarrhoea: Pathogenesis and diagnosis. Vet. J..

[6] Schweizer M., Peterhans E. (2014). Pestiviruses. Annu. Rev. Anim. Biosci..

[7] McClurkin A.W., Littledike E.T., Cutlip R.C., Frank G.H., Coria M.F., Bolin S.R. (1984). Production of cattle immunotolerant to bovine viral diarrhea virus. Can. J. Comp. Med..

[8] Moennig V., Becher P. (2018). Control of Bovine Viral Diarrhea. Pathogens.

[9] Righi C., Petrini S., Pierini I., Giammarioli M., De Mia G.M. (2021). Global Distribution and Genetic Heterogeneity of Border Disease Virus. Viruses.

[10] Becher P., Orlich M., Kosmidou A., König M., Baroth M., Thiel H.J. (1999). Genetic diversity of pestiviruses: Identification of novel groups and implications for classification. Virology.

[11] Schirrmeier H.S.G., Tavella A., Stifter E. Border disease virus infection in cattle-epidemiological and diagnostic impact. Proceedings of the 7th ESVV Pestivirus Symposium.

[12] Nettleton P.F., Gilray J.A., Russo P., Dlissi E. (1998). Border disease of sheep and goats. Vet. Res..

[13] Braun U., Hilbe M., Janett F., Hässig M., Zanoni R., Frei S., Schweizer M. (2015). Transmission of border disease virus from a persistently infected calf to seronegative heifers in early pregnancy. BMC Vet. Res..

[14] Braun U., Hilbe M., Peterhans E., Schweizer M. (2019). Border disease in cattle. Vet. J..

[15] McFadden A.M., Tisdall D.J., Hill F.I., Otterson P., Pulford D., Peake J., Finnegan C.J., La Rocca S.A., Kok-Mun T., Weir A.M. (2012). The first case of a bull persistently infected with Border disease virus in New Zealand. N. Z. Vet. J..

[16] Riitho V., Strong R., Larska M., Graham S.P., Steinbach F. (2020). Bovine Pestivirus Heterogeneity and Its Potential Impact on Vaccination and Diagnosis. Viruses.

[17] Cranwell M.P., Otter A., Errington J., Hogg R.A., Wakeley P., Sandvik T. (2007). Detection of Border disease virus in cattle. Vet. Rec..

[18] Kaiser V., Nebel L., Schüpbach-Regula G., Zanoni R.G., Schweizer M. (2017). Influence of border disease virus (BDV) on serological surveillance within the bovine virus diarrhea (BVD) eradication program in Switzerland. BMC Vet. Res..

[19] Colom-Cadena A.C.O., Rosell R., Fernández-Aguilar X., Blanch-Lázaro B., Tets E., Lavín S., Marco I. (2016). The European hare (Lepus europaeus) as a potential wild reservoir for ruminant pestiviruses. Prev. Vet. Med..

[20] Ridpath J.F., Passler T. (2016). Editorial: Control of Pestivirus Infections in the Management of Wildlife Populations. Front. Microbiol..

[21] Wolff P.L., Schroeder C., McAdoo C., Cox M., Nelson D.D., Evermann J.F., Ridpath J.F. (2016). Evidence of Bovine viral diarrhea virus Infection in Three Species of Sympatric Wild Ungulates in Nevada: Life History Strategies May Maintain Endemic Infections in Wild Populations. Front. Microbiol..

[22] Larska M. (2015). Pestivirus infection in reindeer (*Rangifer tarandus*). Front. Microbiol..

[23] das Neves C.G., Johansson Wensman J., Nymo I.H., Skjerve E., Alenius S., Tryland M. (2019). Pestivirus Infections in Semi-Domesticated Eurasian Tundra Reindeer (*Rangifer tarandus tarandus*): A Retrospective Cross-Sectional Serological Study in Finnmark County, Norway. Viruses.

[24] Wernike K., Beer M. (2024). Comparison of bovine viral diarrhea virus detection methods: Results of an international proficiency trial. Vet. Microbiol..

[25] Bouzalas I.G., Gelasakis A.I., Chassalevris T., Apostolidi E.D., Pappas F., Ekateriniadou L., Boukouvala E., Zdragas A. (2023). Circulation of Pestiviruses in Small Ruminants from Greece: First Molecular Identification of Border Disease Virus. Vaccines.

[26] Wernike K., Beer M. (2022). International proficiency trial for bovine viral diarrhea virus (BVDV) antibody detection: Limitations of milk serology. BMC Vet. Res..

[27] Wernike K., Gethmann J., Schirrmeier H., Schröder R., Conraths F.J., Beer M. (2017). Six Years (2011–2016) of Mandatory Nationwide Bovine Viral Diarrhea Control in Germany—A Success Story. Pathogens.

[28] Beer M., Wernike K., Dräger C., Höper D., Pohlmann A., Bergermann C., Schröder C., Klinkhammer S., Blome S., Hoffmann B. (2017). High Prevalence of Highly Variable Atypical Porcine Pestiviruses Found in Germany. Transbound. Emerg. Dis..

[29] Wylezich C., Papa A., Beer M., Höper D. (2018). A Versatile Sample Processing Workflow for Metagenomic Pathogen Detection. Sci. Rep..

[30] Kumar S., Stecher G., Li M., Knyaz C., Tamura K. (2018). MEGA X: Molecular Evolutionary Genetics Analysis across Computing Platforms. Mol. Biol. Evol..

[31] Tamura K. (1992). Estimation of the number of nucleotide substitutions when there are strong transition-transversion and G+C-content biases. Mol. Biol. Evol..

[32] Spearman C. (1908). The method of right and wrong cases (constant stimuli) without Gauss’s formulae. Br. J. Psychol..

[33] Kärber G. (1931). Beitrag zur kollektiven Behandlung pharmakologischer Reihenversuche. Naunyn-Schmiedebergs Arch. Für Exp. Pathol. Und Pharmakol..

[34] Manual of Diagnostic Tests and Vaccines for Terrestrial Animals, Thirteenth Edition 2024. https://www.woah.org/fileadmin/Home/eng/Health_standards/tahm/3.04.07_BVD.pdf.

[35] Strong R., La Rocca S.A., Ibata G., Sandvik T. (2010). Antigenic and genetic characterisation of border disease viruses isolated from UK cattle. Vet. Microbiol..

[36] Blome S., Beer M., Wernike K. (2017). New Leaves in the Growing Tree of Pestiviruses. Adv. Virus Res..

[37] Balinandi S., Hayer J., Cholleti H., Wille M., Lutwama J.J., Malmberg M., Mugisha L. (2022). Identification and molecular characterization of highly divergent RNA viruses in cattle, Uganda. Virus Res..

[38] Giangaspero M., Harasawa R., Muschko K., Büttner M. (2006). Characteristics of the 5′ untranslated region of wisent (*Bison bonasus*) and reindeer (*Rangifer tarandus*) *Pestivirus* isolates. Vet. Ital..

